# Phase Angle as a Bioelectrical Marker to Identify Sarcopenia

**DOI:** 10.1093/geroni/igaa057.573

**Published:** 2020-12-16

**Authors:** Lisha Hou, Jirong Yue

**Affiliations:** West China Hospital, Chengdu, Sichuan, China

## Abstract

Background: Phase angle (PhA) has been suggested as an indicator of cellular death and nutritional status. We aimed to evaluate the performance of phase angle as a sarcopenia marker among 50 years older and determine the optimal cut-off values. Materials and Methods: A cross section of 4500 with ≥50 years were assessed in terms of sarcopenia with bioelectrical indices. Phase angle can be determined through bioelectrical impedance analysis (In Body 770). Muscle strength and physical function were measured using hand grip and 4 m walking speed. Significant determinants of sarcopenia were further analyzed with multivariate logistic regression analysis. Results: 869 patients (19.31%) were diagnosed with sarcopenia. The average PhA was 5.03 ± 0.64° (Male: 5.31 ± 0.66°; Female: 4.87 ± 0.57°). After adjusting age, gender, race, occupation, BMI, marital status, smoking, drinking, exercise, chronic disease and ADL, phase angle was still independent associated factors with sarcopenia: phase angle (OR=0.25, 95% CI: 0.203-0.308, P < 0.001). Receiver operating characteristic analysis revealed that the optimal phase angle cutoff value to detect sarcopenia was ≤4.9º (AUC=0.768). Conclusions: Bioelectrical phase angle can be an useful bioelectrical marker to identify sarcopenia.

